# Parental oral health knowledge and sociodemographic influences in children aged 2–12 years: a cross-sectional study in Valencia, Spain

**DOI:** 10.1007/s40368-026-01240-9

**Published:** 2026-06-11

**Authors:** C. Borrell-García, S. Sofía Mestanza-Tocto, P. Boo-Gordillo, L. Marqués-Martínez, E. García-Miralles, J. Ignacio Aura-Tormos

**Affiliations:** 1 Faculty of Medicine and Health Sciences, Valencia Catholic University Saint Vincent Martyr, Valencia, Spain; 2 Faculty of Medicine and Health Sciences, University of Valencia, Valencia, Spain

**Keywords:** Parental knowledge, Oral health education, Child oral health, Preventive dentistry, Health literacy

## Abstract

**Purpose:**

The present study aimed to assess parental knowledge of paediatric oral health among parents and guardians of children aged 2–12 years in Valencia, Spain, and to analyse its association with key sociodemographic variables.

**Methods:**

A cross-sectional study was conducted with 208 parents and guardians of children attending a university dental clinic in Spain. A validated self-administered 20-item questionnaire was used to evaluate oral health knowledge. Descriptive statistics and one-way ANOVA with Tukey post hoc tests were applied to compare mean knowledge scores across sociodemographic groups. The significance level was set at *p* < 0.05.

**Results:**

Parental education level was significantly associated with oral health knowledge. Parents with university education (17.01 ± 1.86) and vocational training (16.71 ± 2.09) scored higher than those with secondary school (15.78 ± 2.34), basic education (13.09 ± 2.77), or no education (13.30 ± 2.41). No significant difference was observed by gender (*p* = 0.394). Age group was also associated with knowledge (F(2,195) = 7.55, *p* = 0.001), with the highest scores observed among parents aged 41–50 years (17.45 ± 1.97).

**Conclusions:**

Parental oral health knowledge was associated with educational attainment and age, while no gender differences were observed. These results suggest the value of developing targeted educational approaches for younger parents and those with lower education levels.

## Introduction

Oral health is a fundamental component of overall well-being, particularly in childhood, where it is essential for proper nutrition, speech development, and self-esteem (Agudelo et al. 2023). However, oral diseases, especially dental caries, remain a prevalent global public health issue. The World Health Organization (WHO) highlights oral health as an essential component of overall well-being and a key determinant of quality of life in children. Despite advances in prevention, dental caries remains one of the most prevalent chronic diseases worldwide. Parents and legal guardians play a key role in shaping children’s oral hygiene habits. They are responsible for establishing routines from an early age, supervising daily toothbrushing, influencing diet, and ensuring regular dental check-ups (Brecher and Lewis 2018). In Spain, dental caries continues to affect approximately 30–35% of 12-year-old children according to the most recent national oral health surveys (Mariotti et al. 2025); according to the WHO Global Oral Health Status Report (2022), paediatric oral diseases remain highly prevalent worldwide despite being largely preventable.”

The development of oral pathologies is multifactorial, strongly associated with inadequate oral hygiene, dietary habits high in sugars, and lifestyle factors (Tortora et al. 2023). Dental caries is a highly prevalent, diet-mediated disease that progresses rapidly in children and remains the most common non-communicable condition worldwide (Giacaman et al. 2022; WHO 2022). In paediatric populations, the responsibility for preventing these conditions falls largely on parents or guardians, whose knowledge and daily practices are key determinants of children’s oral health (Mahmoud et al. 2017; Gudipaneni et al. 2021). Parental knowledge strongly aligns with children’s oral hygiene practices (Kumar et al. 2019). Although parental understanding influences preventive behaviours, such as fluoride toothbrushing and dietary control (Goodwin et al. 2024; WHO, 2022), the present study focused exclusively on assessing parental knowledge.

Furthermore, parental education level and socioeconomic background are key factors influencing children’s oral health outcomes. Lower parental education and economic disadvantage are linked to poorer oral health outcomes in children, often due to a combination of less knowledge, unfavourable attitudes, and reduced access to dental services (Deepak and Gautam 2018; Prieto et al. 2023). In Spain, the fact that the public health system typically schedules the first dental examination at age 6 years may inadvertently reinforce a lack of parental awareness regarding the importance of caring for primary teeth from eruption.

Therefore, health education and the promotion of behavioural change from an early age are fundamental to the success of any public health initiative (Al Batayneh et al. 2019; Krishna et al. 2021). Motivational interviewing has also been explored as a strategy to enhance parental engagement in preventive behaviours and improve early oral health outcomes (Naidu et al. 2015). The period between the ages of 2 and 12 years is crucial, as children develop imitative behaviours and are more receptive to adopting lifelong habits. Recent surveys show that both parents and teachers frequently lack adequate oral health knowledge, particularly in early childhood settings (Wang et al. 2024).

Within this context, dentists and parents are considered the primary agents in the prevention of oral diseases, forming a comprehensive health approach (Hernández et al. 2022). School-based oral health education programmes have also demonstrated improvements in children’s knowledge and hygiene behaviours, highlighting the value of structured preventive interventions (Krishna et al. 2021).

Large-scale studies have confirmed that parental education correlates with children’s oral health behaviours and parental knowledge (Chen et al. 2020); however, there is limited evidence on how parental oral health knowledge varies according to sociodemographic factors in the Valencian region, despite its importance for designing effective and targeted preventive programmes. Existing studies in Spain have rarely explored this topic within university dental clinic settings, leaving a gap in understanding the educational needs of parents in this context. While the importance of parental factors is well-established, there remains a need for localised research to inform targeted interventions. Current evidence on the knowledge of parents in the Valencian Community is scarce. Therefore, the aim of this study was to provide a comprehensive overview of these factors among parents and legal guardians of children aged 2–12 years in Valencia, Spain. Specifically, the objectives were to assess the level of parental oral health knowledge, to examine its association with key sociodemographic variables, such as education level, age, and gender, and to determine which caregiver groups may benefit most from targeted oral health education.

## Materials and methods

### Study design

A descriptive, cross-sectional study was conducted in accordance with the Strengthening the Reporting of Observational Studies in Epidemiology (STROBE) guidelines for cross-sectional studies.

### Ethical considerations

This study was conducted in accordance with the ethical principles outlined in the Declaration of Helsinki. Ethical approval was obtained from the Research Ethics Committee of XXXXX. All participants were provided with a detailed information sheet explaining the purpose and procedures of the study and gave written informed consent prior to their inclusion. Data confidentiality and participant anonymity were strictly maintained throughout the study. All identifying information was removed prior to analysis, and digital records were stored securely on password-protected institutional servers with access restricted to the research team, in full compliance with the EU General Data Protection Regulation (GDPR 2016/679).

### Study population and sampling

The study population consisted of 208 parents or legal guardians of school-aged children (aged 2–12 years) who attended the dental clinics of (a university dental clinic in eastern Spain) between September 2023 and March 2024. Participants were recruited using a non-probabilistic convenience sampling approach from among those attending the university dental clinic. The inclusion criteria were: (1) being a parent or legal guardian of a child aged 2–12 years; (2) ability to understand and respond in Spanish; (3) their child being a patient at the university dental clinic; and (4) provision of informed consent. The exclusion criteria were: (1) parents or guardians with physical, sensory, or cognitive impairments that prevented questionnaire completion and (2) individuals who were not the legal guardian of the child attending the clinic.

### Data collection

Parental knowledge was assessed using a previously validated questionnaire originally developed by Cupé and García (2015), consisting of 20 items distributed across three key domains: oral hygiene practices, dietary habits, and preventive measures. Each item offered four possible responses, with only one correct answer, and responses were coded numerically for statistical analysis. The original Spanish version of the instrument underwent content validation by a panel of paediatric dentistry experts to ensure cultural and linguistic appropriateness for the Valencian population. A pilot test involving 20 parents was conducted to evaluate the clarity, reliability, and comprehensibility of the questionnaire, leading to minor wording adjustments prior to its full administration.

Sociodemographic data, including parental age, gender, and educational level, were collected as part of the questionnaire. Educational attainment was categorised into five levels: (1) None—no formal education; (2) GBE/SG—General Basic Education or Secondary Grade; (3) Secondary school—completion of upper secondary education; (4) University—university degree or equivalent; and (5) VT—Vocational Training. These abbreviations are used consistently throughout the manuscript, tables, and figures.

Data collection took place in person at the dental clinics, where trained research staff were available to clarify procedural queries without influencing participants’ responses. Completed questionnaires were entered into a Microsoft Excel database (Microsoft Corp., WA, USA) and subsequently prepared for statistical analysis.

### Sample size calculation

The minimum required sample size was calculated using the standard approach for estimating a single proportion. This calculation was based on an expected prevalence of adequate oral health knowledge of 50% (a conservative estimate used to maximise the sample size), a 95% confidence level (corresponding to a *Z* value of 1.96), and a 7% margin of error. Under these parameters, the minimum sample size was estimated at 196 participants. To account for potential non-response and incomplete data, an additional 10% was added, resulting in a final target sample size of 208 participants. This target was achieved, with 208 parents and legal guardians ultimately included in the study.

### Statistical analysis

Descriptive statistics, including frequencies, percentages, means, and standard deviations, were used to summarise the sociodemographic characteristics of the sample. The distribution of the oral health knowledge score was assessed for normality using the Shapiro–Wilk test. Associations between the dependent variable (oral health knowledge score) and independent variables (educational level, gender, and age group) were examined using independent samples *t* tests and one-way analysis of variance (ANOVA). ANOVA was specifically selected as the most appropriate test for comparing mean scores across more than two independent groups (education levels and age categories). Where the overall ANOVA indicated statistically significant differences, Tukey’s HSD post hoc test was applied to identify pairwise differences when homogeneity of variances was met, while the Games–Howell test was used in cases, where this assumption was violated, as determined by Levene’s test. The level of statistical significance (Type I error) was set at *p* < 0.05 for all analyses.

All statistical analyses were performed using IBM SPSS Statistics for Windows, Version 27 (IBM Corp., Armonk, NY, USA). Figures were generated using the authors’ original dataset exported from SPSS. Generative AI tools were used exclusively to assist in refining graphical formatting and generating plotting code.

## Results

### Participant demographics

A total of 208 parents and legal guardians participated in the study. The majority of respondents were mothers (76.9%), followed by fathers (17.8%) and other caregivers (5.3%). Most participants were aged between 31 and 40 years (55.8%), while 33.2% were between 20 and 30 years, and 10.6% were between 41 and 50 years. Only one participant (0.5%) was aged between 51 and 60 years.

Regarding educational attainment, secondary school (38.9%) and university education (32.2%) were the most frequent categories, followed by basic education (17.3%), vocational training (6.7%), and no formal education (4.8%). Detailed sociodemographic characteristics are presented in Table 1.

**Table 1:** Frequencies and percentages for sociodemographic variables (*N* = 208)

Variable	Category	Frequency *N* (%)
Distribution of Respondents	Father	37 (17.8)
	Mother	160 (76,9)
	Guardian/Caregiver	11 (5.3)
Parental education level	None	10 (4.8)
	Basic education/School graduate	36 (17.3)
	Vocational training	14 (6.7)
	Secondary school	81 (38.9)
	University	67 (32.2)
Age of the respondent in years (range)	20 – 30	69 (33.2)
	31 – 40	116 (55.8)
	41 – 50	22 (10.6)
	51 – 60	1 (0.5)

### Parental oral health knowledge according to educational level

**Fig. 1:**
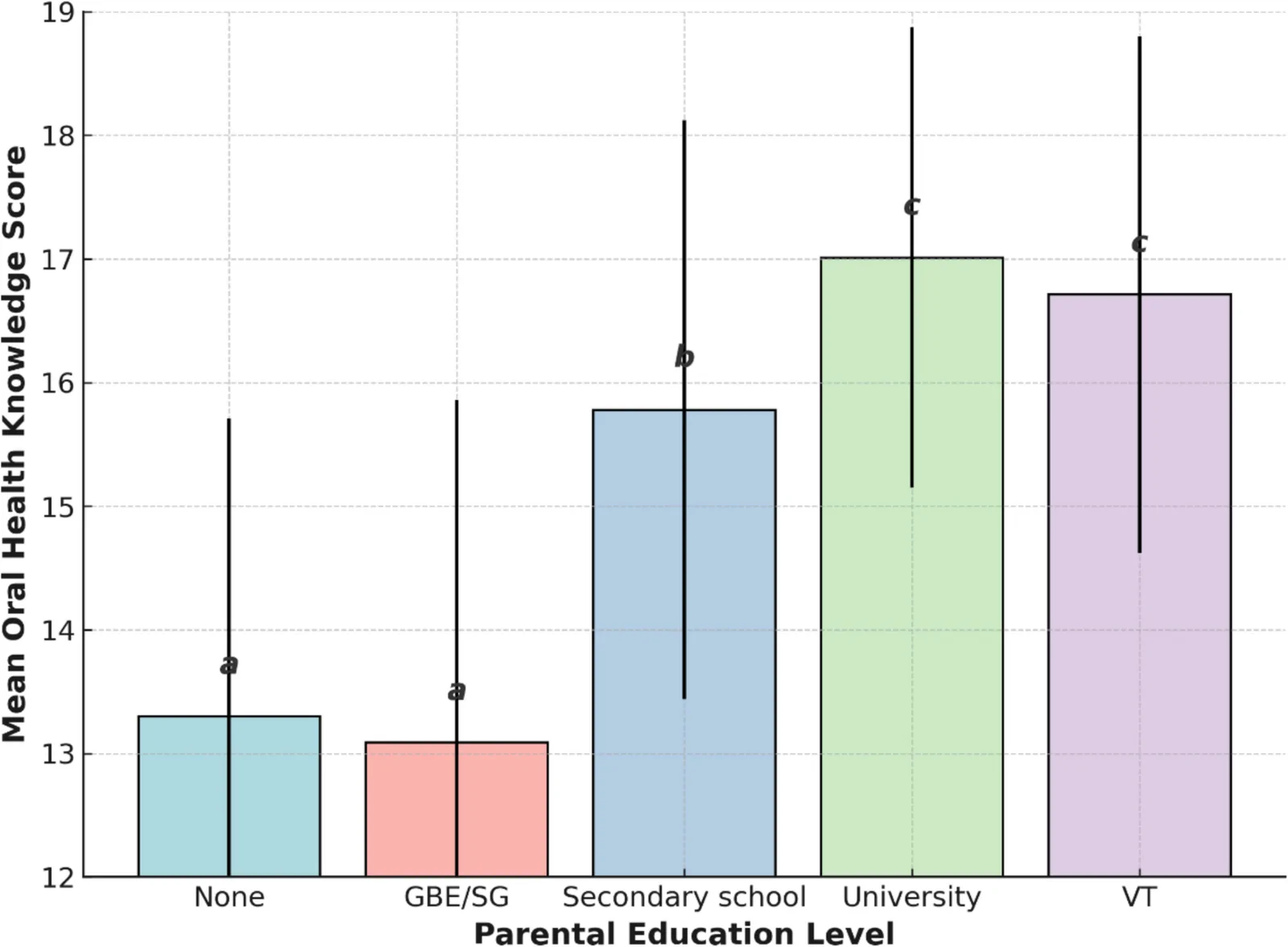
Mean parental oral health knowledge scores by education level

A statistically significant association was observed between parental education level and overall oral health knowledge (F(4,202) = 20.82, *p* < 0.001). Knowledge scores increased progressively with higher levels of educational attainment, with university and vocational training groups achieving the highest values and basic or no-education groups the lowest (Fig. 1).

Error bars represent ± 1 standard deviation. Bars sharing the same superscript letter (a, b, c) are not statistically significantly different (Tukey’s HSD, *p* < 0.05).

P*ost hoc* comparisons showed an educational gradient, with statistically significantly higher scores consistently observed at higher educational levels (Table 2).

**Table 2:** Tukey post hoc pairwise comparisons between parental education levels for oral health knowledge

Comparison	Mean Diff. (MD)	*p*	95% CI Lower	95% CI Upper
None vs. GBE/SG	0.21	0.999	− 2.02	2.45
None vs. Secondary school	− 2.48	0.011	− 4.57	− 0.39
None vs. University	− 3.71	< 0.001	− .83	− 1.60
None vs. VT	− 3.41	0.003	− 6.00	− 0.83
GBE/SG vs. Secondary school	− 2.69	< 0.001	− 3.95	− 1.43
GBE/SG vs. University	− 3.93	< 0.001	− 5.23	− 2.63
GBE/SG vs. VT	− 3.63	< 0.001	− 5.60	− 1.66
Secondary vs. University	− 1.24	0.010	− 2.27	− 0.21
Secondary vs. VT	− 0.94	0.610	− 2.74	0.87
University vs. VT	0.30	0.991	− 1.54	2.13

Comparison codes: *GBE/SG* General Basic Education/Secondary Grade; *VT* Vocational Training; *MD* Mean difference

### Parental oral health knowledge according to gender

An independent samples *t* test showed no statistically significant differences in parental oral health knowledge between fathers and mothers (t(194) = 1.14, *p* = 0.257). Mean scores for both groups were comparable, as shown in Table 3.

**Table 3:** Independent samples *t* -test comparing oral health knowledge by respondent gender

DV	Respondent	N	Mean	SD	t(df)	*p*	MD	95% CI Lower	95% CI Upper
Oral Health	Father	37	16.14	2.50	1.14	0.257	0.55	− 0.41	1.51
	Mother	159	15.58	2.69					

### Parental oral health knowledge according to age group

A statistically significant association was found between age group and overall oral health knowledge (F(2,195) = 7.55, *p* = 0.001). Parents aged 41–50 years achieved the highest knowledge scores (Table 4).

**Table 4:** Descriptive statistics and one-way ANOVA for oral health knowledge by age group

Variable	Category (age group)	Mean	SD	N	Levene’s F	*p*	ANOVA F(2,195)	*p*	Partial η^2^	1-*β*
Oral Health	20–30	15.00	2.92	66	3.874	0.022	7.55	0.001	0.073	0.942
	31–40	15.74	2.45	109						
	41–50	17.45	1.97	22						

P*ost hoc* comparisons confirmed that the 41–50 year age group scored significantly higher than both younger groups, while no statistically significant difference was observed between the 20–30 year and 31–40 year age groups (Table 5).

**Table 5:** Games–Howell post hoc pairwise comparisons between age groups for oral health knowledge

Comparison (age group)	Mean Diff. (MD)	*p*	95% CI Lower	95% CI Upper
20–30 vs. 31–40	− 0.74	0.201	− 1.77	0.28
20–30 vs. 41–50	− 2.45*	< 0.001	− 3.79	− 1.12
31–40 vs. 41–50	− 1.71	0.003	− 2.89	− 0.54

## Discussion

The present study provides a comprehensive analysis of the knowledge concerning oral health amongst parents and caregivers of school-aged children in eastern Spain. The findings illuminate significant relationships between sociodemographic factors and parental oral health knowledge, offering valuable insights for public health strategy.

The most salient finding of the present research is the strong, positive association between parental education level and oral health knowledge; demonstrating that parents with higher educational qualifications, particularly at the university and vocational training levels, possess significantly greater knowledge than those with only basic or no formal education. This gradient effect underscores education as a key social determinant of health literacy and is consistent with previous research by Teixeira et al. (2011) and Prieto et al. (2023), who similarly reported that higher maternal education levels were correlated with superior oral health knowledge and better child outcomes. This consistency suggests that improving general educational attainment could yield significant collateral benefits for paediatric oral health.

A notable strength of the present study is that it provides the first Valencia-specific data on parental oral health knowledge collected within a university dental clinic setting. This local evidence is particularly valuable, as sociodemographic patterns and access to preventive services vary across regions, and no prior studies have documented these associations in the Valencian Community.

These findings are consistent with wider European evidence showing persistent educational and socioeconomic inequalities in parental oral health knowledge and children’s oral health outcomes. Similar patterns have been reported in recent studies from Italy (Capurro et al. 2025), the UK (Rouxel & Chandola 2018), and primary care settings in Europe and South America, where lower health literacy is associated with poorer dental outcomes (Mialhe et al. 2022). Such findings underline the need for coordinated public health strategies across Europe to strengthen caregiver health literacy, particularly among populations with lower educational attainment. In Spain, this reinforces the importance of integrating preventive oral health education into primary care and school-based programmes, aligning national efforts with broader European public health priorities.

Contrary to some previous studies, the present data indicate that older parents, particularly those in the 41–50 year age range, possess significantly greater oral health knowledge than their younger counterparts. This contrasts with the findings of Cupé and García (2015) and Rengifo et al. (2019), who reported that younger mothers demonstrated higher knowledge levels. This discrepancy may be attributed to life experience, cumulative exposure to health information over time, or cohort effects specific to the Valencian context. It suggests that younger, first-time parents may represent a key target group for educational interventions, as they are at a critical juncture for establishing family health behaviours.

A noteworthy finding was the absence of a statistically significant difference in knowledge scores between mothers and fathers. This challenges the assumptions of some previous studies, such as Valladares et al. (2023), who found fathers to have greater knowledge, and Vaca (2024), who reported the opposite. The present results suggest that in the contemporary context, gender may be a less reliable predictor of oral health knowledge than factors, such as education or age. This underscores the importance of developing educational initiatives that are inclusive and targeted at all caregivers, regardless of gender.

The influence of socioeconomic conditions, as highlighted by Deepak and Gautam (2018), is implicitly reflected in our findings through the education variable. Lower knowledge levels among less-educated parents likely intersect with economic disadvantage, potentially leading to inadequate dental care and higher consumption of cariogenic foods, as observed by Mahmoud et al. (2017). Furthermore, the present results support the conceptual model that parental knowledge is a foundational element for the development of self-efficacy and planning capabilities, which are crucial for supervising daily health behaviours like toothbrushing, as emphasised by Gudipaneni et al. (2021).

A primary strength of the present study is its focus on an underexplored yet critical area of paediatric health, utilising a validated instrument and a robust sample size. However, several limitations must be acknowledged. The cross-sectional design precludes the establishment of causal relationships, and the reliance on self-reported data may introduce social desirability bias. A further limitation relates to the use of convenience sampling within a university dental clinic, which may restrict the generalisability of the findings. Although this approach enabled efficient access to the target population, the sample may not fully represent families who do not routinely attend dental services or those from different socioeconomic contexts. Future research should employ longitudinal designs to track how parental knowledge evolves and influences children’s oral health outcomes over time, and probabilistic sampling strategies could help validate and extend these findings. Qualitative studies may also provide deeper insight into the barriers and facilitators that parents face in implementing optimal oral health practices.

## Conclusions

The present study highlights the relevance of parental education and life stage in shaping oral health knowledge during childhood. Rather than pointing to gender-related differences, the findings suggest that disparities in knowledge are more strongly driven by educational background and age-related experience.

## Data Availability

The data that support the findings of this study are available from the corresponding author upon reasonable request.

## Declarations

### Additional information

Generative AI tools (ChatGPT, OpenAI) were used only to assist with plotting code and graphical formatting for Fig. 1 using the authors’ original dataset. No AI-generated data, analyses, images, or scientific content were produced, and all final decisions were made by the authors.
